# HLA Alleles Associated with Delayed Progression to AIDS Contribute Strongly to the Initial CD8^+^ T Cell Response against HIV-1

**DOI:** 10.1371/journal.pmed.0030403

**Published:** 2006-10-31

**Authors:** Marcus Altfeld, Elizabeth T Kalife, Ying Qi, Hendrik Streeck, Mathias Lichterfeld, Mary N Johnston, Nicole Burgett, Martha E Swartz, Amy Yang, Galit Alter, Xu G Yu, Angela Meier, Juergen K Rockstroh, Todd M Allen, Heiko Jessen, Eric S Rosenberg, Mary Carrington, Bruce D Walker

**Affiliations:** 1 Partners AIDS Research Center, Infectious Disease Unit, Massachusetts General Hospital and Division of AIDS, Harvard Medical School Boston, Boston, Massachusetts, United States of America; 2 Basic Research Program, SAIC-Frederick, Laboratory of Genomic Diversity, National Cancer Institute, Frederick, Maryland, United States of America; 3 Gemeinschaftspraxis Jessen, Berlin, Germany; 4 Department of Internal Medicine, University of Bonn, Bonn, Germany; 5 Howard Hughes Medical Institute, Chevy Chase, Maryland, United States of America; University of Wisconsin, United States of America

## Abstract

**Background:**

Very little is known about the immunodominance patterns of HIV-1-specific T cell responses during primary HIV-1 infection and the reasons for human lymphocyte antigen (HLA) modulation of disease progression.

**Methods and Findings:**

In a cohort of 104 individuals with primary HIV-1 infection, we demonstrate that a subset of CD8^+^ T cell epitopes within HIV-1 are consistently targeted early after infection, while other epitopes subsequently targeted through the same HLA class I alleles are rarely recognized. Certain HLA alleles consistently contributed more than others to the total virus-specific CD8^+^ T cell response during primary infection, and also reduced the absolute magnitude of responses restricted by other alleles if coexpressed in the same individual, consistent with immunodomination. Furthermore, individual HLA class I alleles that have been associated with slower HIV-1 disease progression contributed strongly to the total HIV-1-specific CD8^+^ T cell response during primary infection.

**Conclusions:**

These data demonstrate consistent immunodominance patterns of HIV-1-specific CD8^+^ T cell responses during primary infection and provide a mechanistic explanation for the protective effect of specific HLA class I alleles on HIV-1 disease progression.

## Abstract 2

A subset of CD8^+^ T cell epitopes within HIV-1 are consistently targeted early after infection. This could explain some of the protective effect of certain HLA class I alleles on HIV-1 disease progression.

## Editors' Summary

**Background.:**

Nearly 15,000 new HIV infections occur each day. There is no cure for HIV, and the treatments currently used to prevent people with HIV from dying are expensive and unavailable to many who need them. There is also no vaccine to prevent HIV. An effective vaccine would somehow induce the immune system to prevent the virus from reaching destructive levels in the body, but how to design such a vaccine is unknown.

In most people infected with HIV, the immune system doesn't keep the AIDS virus in check over the long term. It has been known for a long time, however, that the body somehow brings the virus under control within a few weeks following infection, after which, in the absence of treatment, the amount of virus gradually increases again over time. Exactly why the amount of virus drops after initial infection is not fully understood, but there is good evidence that the white blood cells called CD8 T lymphocytes, which can kill other cells infected with viruses, are at least partially responsible for initially bringing HIV infection under control.

In order for a CD8 T lymphocyte to recognize and kill an infected cell, that cell has to display some part of the infecting virus on its surface. There are many possible fragments of HIV that can activate CD8 T cells, although some of these fragments appear more effective than others at provoking a strong killer response. Also, in order to activate CD8 T cells the viral fragments must bind to and be presented by a particular kind of protein called HLA on the surface of the infected cells. There are hundreds of varieties of HLA in the human population, allowing our immune systems to recognize many parts of many different viruses. (Each person can have up to six different kinds of HLA class I on the surface of his or her cells). A few specific types of HLA have been found to provide some advantage in keeping the AIDS virus under control, possibly because they present fragments of the virus that are particularly good at activating CD8 T cells.

**Why Was This Study Done?:**

The researchers wanted to find out whether specific HLA types and specific protein fragments (peptides) of the AIDS virus are particularly important in helping CD8 T cells control HIV. Specifically, they wanted to find out the very earliest protein fragments recognized, since these might be particularly important in keeping the virus in check. They also wanted to see if these particular HLA-peptide combinations might affect the long-term health of people with HIV infection. Finding specific combinations of peptide and HLA that give rise to strong control of HIV could help in the design of an effective AIDS vaccine.

**What Did the Researchers Do and Find?:**

The researchers studied CD8 T cells in blood samples from 104 people in the early stages of HIV infection. They used DNA analysis to determine which HLA types were present in each participant, and then chose, from among 173 different protein fragments of HIV, the peptides that are known to bind to and be presented by the participant's HLA types. The ability of these peptides to activate the participant's CD8 T cells was measured in the laboratory.

These studies found that for many types of HLA, there were a few specific viral peptides that triggered most of the CD8 T cell activity found in early HIV infection, when the amount of virus in the blood is being lowered by this response. A few types of HLA were found to contribute more strongly than others to CD8 T-cell activity in early HIV infection. These same types of HLA are also found in people who tend to remain healthier for a longer time after becoming infected with HIV.

**What Do These Findings Mean?:**

This study provides evidence that ability of CD8 T cells to keep HIV under control in the first few months following infection depends on a person's HLA composition, and that this early CD8 T cell activity sets the stage for the long-term balance between the body and the virus. Knowing the particular peptide–HLA combinations that dominate the early immune response (when the immune system appears to be bringing the virus under control) might be of use in designing an HIV vaccine. Because this study was done in people already infected with HIV, however, it remains unclear whether a vaccine based on this knowledge would actually prevent new HIV infection or improve health after infection, even in people with “good” HLA types.

**Additional Information.:**

Please access these Web sites via the online version of this summary at http://dx.doi.org/10.1371/journal.pmed.0030403.

- The UCSF Center for HIV Information's HIV InSite includes resources on HIV immunology and vaccine development
- The Los Alamos Natural Laboratory HIV Molecular Immunology Database contains information for researchers on HIV peptides, HLA, and CD8 T cell responses
- The International AIDS Vaccine Initiative (IAVI) Web site provides policy and scientific information on the global AIDS vaccine effort
- The Massachuetts General Hospital Web site contains information about their HIV-1 research programs

## Introduction

The majority of individuals infected with HIV-1 develop an acute viral syndrome within 7–21 days of infection, characterized primarily by fever, lymphadenopathy, and cutaneous rash in the presence of very high levels of HIV-1 replication [1,2]. Both innate and adaptive immune responses, including natural killer cell responses, HIV-1-specific CD4^+^ and CD8^+^ T cell responses, and neutralizing antibodies have been associated with the subsequent resolution of the clinical symptoms and the decline of HIV-1 RNA levels to so-called viral set point levels. In particular, the first appearance of HIV-1-specific CD8^+^ T cells in the peripheral blood has been shown to be temporally associated with the initial decline of HIV-1 viremia during primary infection [3,4], suggesting a crucial role of these early virus-specific T cells in the control of viral replication. This is further supported by the lack of decline in viral replication in simian immunodeficiency virus-infected macaques depleted of CD8^+^ lymphocytes [5–7] and the selection of viral strains containing sequence variations within targeted CD8^+^ T cell epitopes during this early phase of infection [8–11], indicative of HLA class I-restricted immune selection pressure on the virus.

Despite this presumed immune-mediated decline in acute viremia, HIV-1-specific CD8^+^ T cell responses in primary infection are of lower magnitude and more narrowly directed against a limited number of epitopes than are HIV-1-specific CD8^+^ T cell responses detected in chronic infection [12–15], indicating that the quality and specificity, rather than the quantity, of virus-specific CD8^+^ T cell responses may be associated with the initial control of viral replication [16–20]. However, very little is known about the individual epitopes targeted during primary HIV-1 infection and their immunodominance pattern, as these studies are complicated by the high HLA class I diversity in humans, requiring large numbers of participants with primary infection to draw meaningful conclusions. To date, studies of the specificity of HIV-1-specific CD8^+^ T cell responses during primary HIV-1 infection have been limited in size, focused on individuals expressing specific HLA alleles of interest, or assessed the protein specificity of these CD8^+^ T cells without determining the individual targeted epitopes in the context of the restricting HLA class I molecules [12–15,21–25].

Here, we describe the characterization of HIV-1-specific CD8^+^ T cell responses on the single-epitope level in a cohort of 104 individuals identified during primary HIV-1 infection. The aim of the study was to identify the immunodominant CD8^+^ T cell epitopes within HIV-1 that are targeted during primary HIV-1 infection, and to assess the contribution of CD8^+^ T cell responses restricted by the individual HLA class I molecules to the total virus-specific CD8^+^ T cell response early in infection.

## Methods

### Study Participants

A total of 104 HIV-1-infected individuals were enrolled in this study at the Massachusetts General Hospital in Boston, Massachusetts, United States, and a private medical clinic (Jessen-Praxis) in Berlin, Germany. Of this group, 69 (66%) individuals were identified during acute HIV-1 infection, as defined by either a negative HIV-1 p24 ELISA or an evolving HIV-1 Western blot (fewer than three bands), and the remaining 35 (34%) were identified within the first 6 mo of HIV-1 infection, as defined by a negative HIV-1 p24 ELISA during the past 6 mo or a negative detuned HIV-1 ELISA at the time of enrollment. The majority of study participants were men who have sex with men (98 [94%]) and individuals of Northern European descent (83 [80%]). The average viral load at presentation was 3,527,188 HIV-1 RNA copies/ml (range 50–84,200,000 copies) and the average CD4^+^ T cell count was 520 cells/μl (range 42–1,334). The majority of individuals (88 [85%]) initiated HAART during primary infection. The assessment of HIV-1-specific CD8^+^ T cell responses was performed on frozen peripheral blood mononuclear cell (PBMC) samples collected 8 wk (± 10 d) following initial presentation. This time point was chosen because previous studies had demonstrated that a substantial subset of individuals with acute HIV-1 infection have no detectable or only very weakly detectable HIV-1-specific CD8^+^ T cell responses at presentation, and that the HAART-induced decline in virus-specific T cell responses occurs after 8 wk of treatment in individuals treated with HAART during acute or early HIV-1 infection [12]. The study was approved by the respective institutional review boards and was conducted in accordance with human experimentation guidelines of the Massachusetts General Hospital, and all study participants provided informed consent prior to enrollment in the study.

### HLA Typing

High- and intermediate-resolution HLA class I typing was performed by sequence-specific PCR according to standard procedures. DNA was extracted from PBMCs using the Puregene DNA Isolation Kit for blood (Gentra Systems, Minneapolis, Minnesota, United States).

### IFN-γ Enzyme-Linked Immunosorbent Spot Assay

HIV-1-specific CD8^+^ T cell responses were quantified by IFN-γ enzyme-linked immunosorbent spot (ELISPOT) assay, using a panel of 173 peptides corresponding to described optimal clade B cytotoxic T lymphocyte epitopes [26]. PBMCs were plated at 100,000 cells per well with peptides at a final concentration of 10^−5^ molar in 96-well plates and processed as described [12]. PBMCs were incubated with media alone (negative control) or PHA (positive control). The number of specific IFN-γ secreting T-cells were counted using an automated ELISPOT reader (AID, Strassberg, Germany), calculated by subtracting the average negative control value and expressed as spot-forming cells (SFCs)/10^6^ input cells. Negative controls were always 30 SFCs/10^6^ input cells or fewer. A response was considered positive at 50 SFCs/10^6^ input cells or more and when the total number of spots were at least three times greater than the mean number of spots in the negative control wells.

### Hazard Ratios for Disease Outcomes

The hazard ratios (HRs) for the various HLA alleles were determined previously using Cox model analyses [27–30]. Briefly, 1,217 HIV-1-infected individuals for whom the dates of seroconversion were known were derived from four cohorts: the Multicenter AIDS Cohort Study (MACS, *n* = 522), the Multicenter Hemophilia Cohort Study (MHCS, *n* = 322), the San Francisco City Clinic Cohort (SFCCC, *n* = 87), and the AIDS Linked to Intravenous Experience (ALIVE, *n* = 286) study. Survival analyses were performed on seroconverters from all the cohorts combined and included all patients without regard to racial group. Four AIDS-related outcomes were considered end points of survival analysis: a CD4^+^ T lymphocyte count of less than 200/mm^3^ (hereafter termed “CD4 <200”), progression to AIDS according to the 1993 definition of the CDC (“AIDS 1993”), progression to AIDS according to the more stringent 1987 CDC definition (“AIDS 1987”), and death. Participants in the Cox model analyses were stratified by race and age at seroconversion.

### Statistical Analysis

Statistical analysis and graphical presentation was done using SigmaPlot 5.0 (SPSS, Chicago, Illinois, United States). Results are given as mean ± standard deviation (SD) or median with range. Statistical analysis of significance (*p*-values) was based on two-tailed t-tests and linear regression analysis.

## Results

### Preferential Recognition of a Subset of HIV-1-Specific CD8^+^ T Cell Epitopes during Primary Infection

In order to identify the individual HIV-1-specific CD8^+^ T cell epitopes targeted during primary infection, as well as their relative contribution to the entire detectable HIV-1-specific CD8^+^ T cell response, cryopreserved PBMCs from 104 study participants were tested for responses to peptides corresponding to previously described epitopes for each person's respective HLA class I allotypes [26] using an IFN-γ ELISPOT assay. The analysis in this study was restricted to HLA class I alleles that were expressed in at least three participants, and for which at least three optimal CD8^+^ T cell epitopes had been described. Table 1 lists the frequencies of these 21 qualifying HLA class I alleles expressed in the study population, as well as the number of epitopes tested for each HLA allele and the percentage of individuals with detectable CD8^+^ T cell responses to the tested epitopic peptides restricted by the designated allele. The sequences and HLA class I restrictions of the tested HIV-1 peptides are listed in Table 2.

**Table 1:**
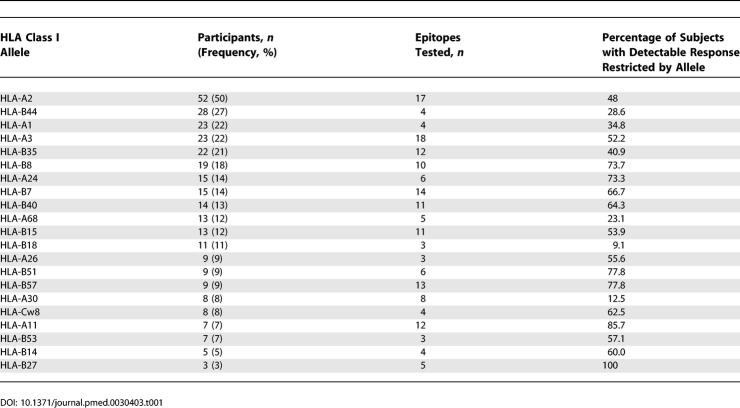
HLA Class I Frequencies in Study Population

**Table 2:**
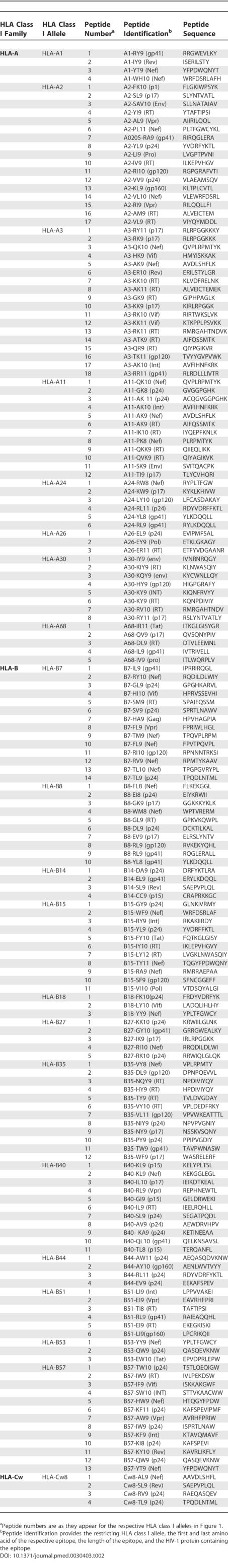
HIV-1-Specific CD8^+^ T Cell Epitopes Tested

**Figure 1:**
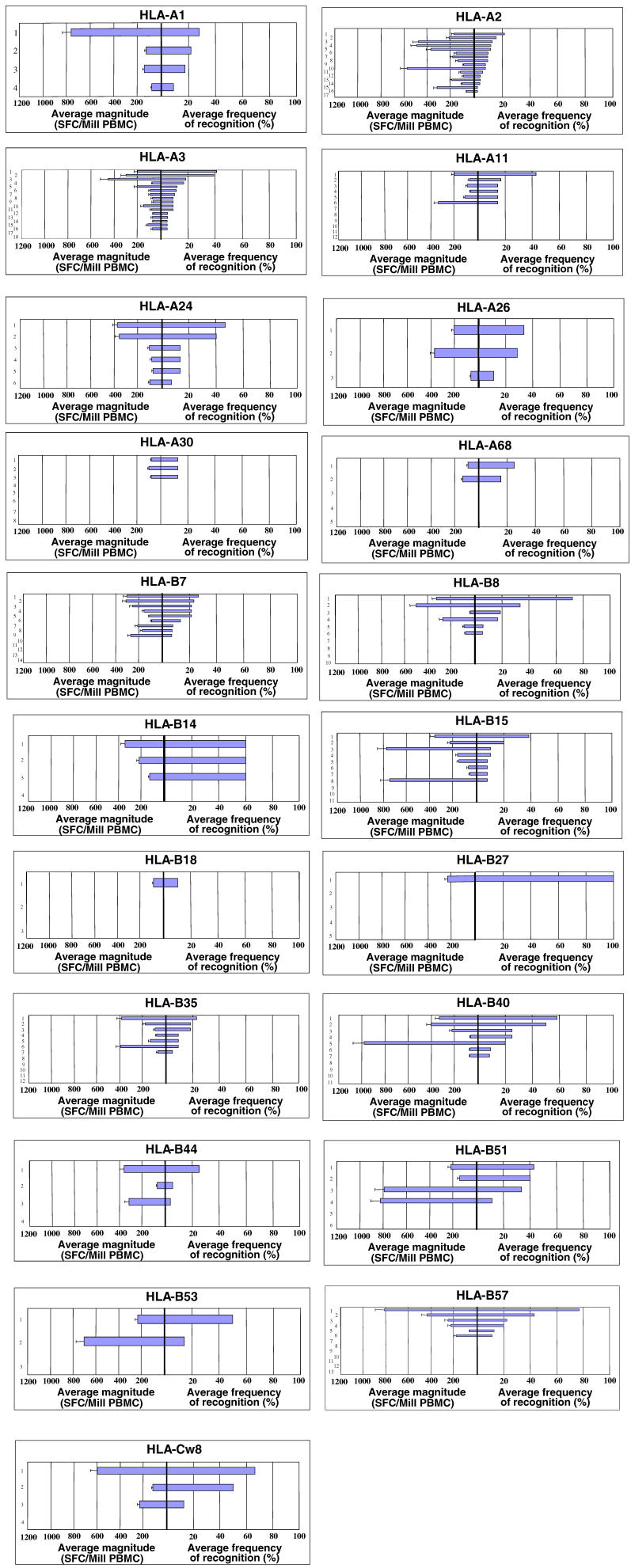
Immunodominance Patterns for HIV-1-Specific CD8^+^ T Cell Responses Restricted by Individual HLA Class I Alleles Peptides corresponding to described optimal HIV-1-specific CD8^+^ T cell epitopes were tested in all study participants expressing the respective HLA class I allele. The average magnitude of CD8^+^ T cell responses specific for each tested peptide, given as SFCs per million input PBMCs (SFC/Mill PBMC), are shown as bars in the left part of each graph. The percentage of participants expressing the respective allele that had a detectable peptide-specific CD8^+^ T cell response are shown as bars on the right part of each graph. Epitopes are aligned for each HLA class I allele according to their frequency of recognition from top to bottom. The peptide number corresponds to the peptide sequence listed for each HLA allele and number in Table 2. Data are shown only for HLA class I alleles that were expressed in at least three individuals, and for which at least three HIV-1-specific optimal CD8^+^ T cell epitopes had been defined.

Each study participant was tested for CD8^+^ T cell responses against a median of 31 epitopic peptides (range 5–53) reported to be recognized in the context of their expressed HLA class I alleles, and a median of 3.8 peptides (range 0–20) induced significant IFN-γ production of CD8^+^ T cells per participant. The number of epitopes tested per HLA class I allele did not correlate with the percentage of individuals that had detectable responses restricted by that allele (Table 1; *r* = 0.29, *p* = 0.2), demonstrating that the differences in the number of tested peptides per allele did not represent a major confounding factor for the analysis. Figure 1 summarizes the magnitude of CD8^+^ T cell responses to the individual HIV-1-specific CD8^+^ T cell epitopes tested (bars on left of each graph), as well as the frequency by which they were recognized in individuals expressing the respective HLA class I allele (bars on right of each graph). The data demonstrate clear immunodominance patterns for the majority of HLA class I alleles, with individual epitopes targeted frequently by individuals expressing the respective allele, while other epitopes restricted by the same allele were rarely recognized in primary infection (Figure 1 and Table 2).

A subanalysis of immunodominance patterns was performed between individuals identified during acute (69/104) and early (35/104) HIV-1 infection for those HLA class I alleles for which at least three individuals were present in both groups (HLA-A1, -A2, -A3, -A24, -A68, -B7, -B8, -B15, -B35, -B40, -B44, and -B53, as well as HLA-B57 and -Cw8, for which only two participants were included in the early infection group). This analysis demonstrated that the immunodominant HIV-1-specific CD8^+^ T cell responses were identical in both acute and early infection for HLA-A1, -A2, -A24, -B8, -B15, -B40, -B44, -B53, -B57, and -Cw8. For HLA-A68 and -B35, no HIV-1-specific CD8^+^ T cell responses were detected in at least one of the two groups. A switch in the immunodominant HIV-1-specific CD8^+^ T cell responses between acute and early infection was only observed in the case of responses restricted by HLA-A3 and HLA-B7. In HLA-A3, the overlapping p17 Gag epitopes RK9 and RY10 represented the dominant epitopes in acute infection (46% and 48% of participants targeted these epitopes, respectively), but were targeted only by 29% and 27% of those identified during early infection. In contrast, the Nef epitope QK10 was targeted by 15% of individuals identified during acute infection, but the immunodominant epitope in persons with early infection (43%). This switch in immunodominance may be the result of sequence evolution within the RK9/RY10 epitopes, or their flanking regions, as described previously [24]. Finally, the HLA-B7 restricted Env epitope IL9 was dominant in patients identified in acute infection (40%), but less frequently recognized in those with early infection (20%), while the subdominant epitope-specific CD8^+^ T cell response directed against the RT SM9 epitope (10% in acute infection) was the most frequently detected response in the patients identified during early infection (50%). However, only five participants with early infection, compared to ten with acute infection, were present in our study cohort, and the differences in immunodominance for these two epitopes did not reach statistical significance (B7-IL9, *p* = 0.6; B7-SM9, *p* = 0.1). The results from this subanalysis demonstrate that, although there was some evidence of ongoing evolution of individual immune responses from acute to early infection, the immunodominance patterns of HIV-1-specific CD8^+^ T cell responses did not differ significantly between the 69 individuals identified during acute HIV-1 infection and the 35 persons identified during early infection.

**Figure 2:**
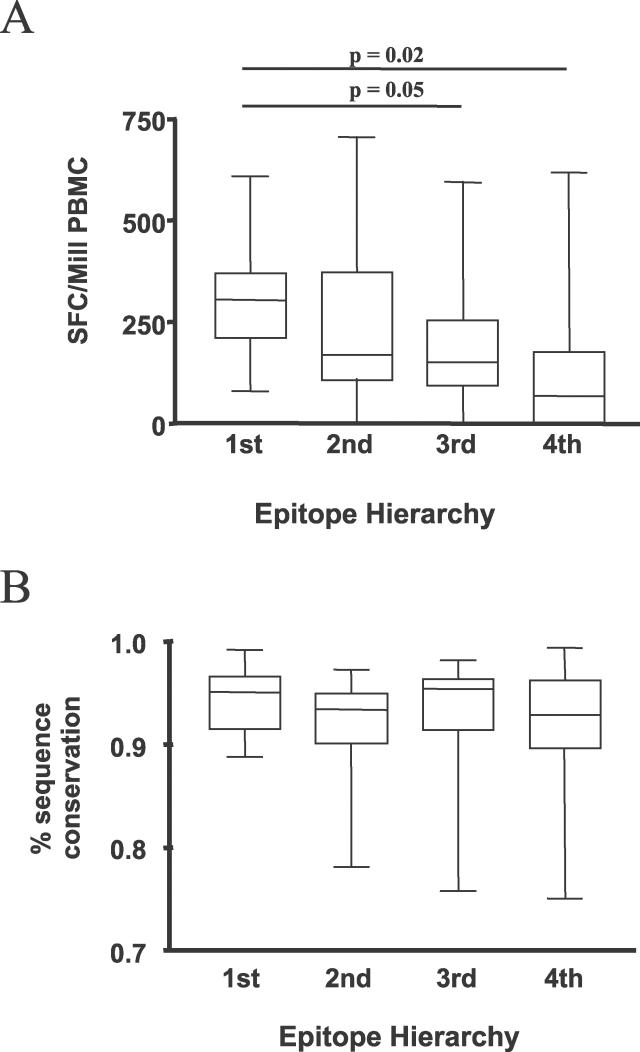
CD8^+^ T Cell responses Directed against the Most Frequently Recognized CD8^+^ T Cell Epitopes Were Also of the Highest Magnitude (A) For each HLA class I allele studied, the four most frequently targeted HIV-1-specific CD8^+^ T cell epitopes were listed according to their hierarchy (for HLA class I alleles with only three described epitopes, the fourth value was excluded from the analysis). The average magnitudes of CD8^+^ T cell responses directed against the 1st, 2nd, 3rd, and 4th most frequently targeted epitope for each allele were calculated (given as SFCs per million input PBMCs [SFC/Mill PBMC]) and are shown as box plots. The average magnitude of the most frequently targeted HIV-1-specific CD8^+^ T cell epitopes restricted by each allele were significantly higher than the average magnitude of the 3rd and 4th most frequently targeted epitopes, and the respective *p*-values are provided above the box plot. (B) The sequence conservation within targeted CD8^+^ T cell epitopes does not contribute significantly to the observed immunodominance patterns of HIV-1-specific CD8^+^ T cell response in primary infection. For each HLA class I alleles studied, the four most frequently targeted HIV-1-specific CD8^+^ T cell epitopes were listed according to their hierarchy (for HLA class I alleles with only three described epitopes; the fourth value was excluded from the analysis). The average sequence conservations, in comparison to HIV-1 clade B sequences published in the Los Alamos Database, of the 1st, 2nd, 3rd, and 4th most frequently targeted epitope for each allele were calculated (given as percent sequence conservation) and are shown as box plots. The average percentage of sequence conservation of the four most frequently targeted HIV-1-specific CD8^+^ T cell epitopes restricted by each allele did not differ significantly.

In addition to the presence of distinct immunodominant epitope-specific CD8^+^ T cell responses during primary HIV-1 infection, there was a significant tendency for the most frequently recognized epitopes to represent the epitopes inducing the strongest CD8^+^ T cell responses in terms of magnitude (Figure 2A). Interestingly, ten epitopes restricted by seven distinct alleles were recognized by CD8^+^ T cells of more than half of individuals expressing the respective HLA class I molecule, and all except one HLA-Cw8-restricted epitope in HIV-1 Nef were restricted by HLA-B alleles, in line with previous data describing a dominant influence of HLA-B alleles in restricting CD8^+^ T cell responses in HIV-1 clade C infection [31]. Taken together, these data demonstrate that consistent immunodominance patterns can be identified for HIV-1-specific CD8^+^ T cell epitopes restricted by common HLA class I molecules in primary HIV-1 infection, and that the preferentially targeted epitopes early after infection are predominantly restricted by HLA-B alleles.

### No Differences in Sequence Diversity between Frequently and Rarely Targeted CD8^+^ T Cell Epitopes in Primary HIV-1 Infection

We next analyzed the sequence diversity in the frequently targeted epitopes compared to those that were restricted by the same HLA class I allele but rarely targeted, since we had demonstrated previously for selected HIV-1-specific CD8^+^ T cell epitopes that their sequence heterogeneity in the circulating HIV-1 strains can be an important determinant for the ability of the immune system to recognize these epitopes [24,32–35], whereas some epitopes are only targeted later in infection despite the presence of the original wild-type sequence in the initial infecting viral strain [21]. This more comprehensive analysis in 104 HIV-1 infected individuals identified during primary infection here demonstrates that the sequence variability of HIV-1 does not represent a major determinant for the immunodominance patterns of detectable epitope-specific CD8^+^ T cell responses directed against the virus. The degree of conservation between the immunodominant epitopes targeted by each studied allele and subdominant epitopes did not differ significantly (Figure 2B), suggesting that factors other than just the sequence variability of the epitope are important in determining the frequency that epitopes will be targeted.

### Immunodomination of Selected HIV-1-Specific CD8^+^ T Cell Responses Restricted by Individual HLA Class I Alleles

Each study participant, if fully heterozygous, expressed six distinct HLA class I alleles, and each of these alleles can contribute to the total CD8^+^ T cell response by presenting epitopes on the surface of infected cells. We therefore assessed the contribution of each individual HLA allele to the total HIV-1-specific CD8^+^ T cell response detected in individuals expressing the respective allele. As an example, for each HLA-A1-positive person the relative contribution of CD8^+^ T cell responses against HLA-A1-restricted epitopes to the total HIV-1-specific CD8^+^ T cell response against all tested HIV-1 epitopes for the participant's HLA allotype was determined; subsequently, the average contribution of HLA-A1-restricted responses was calculated for all HLA-A1-positive participants.

Overall, although the differences did not reach statistical significance, there was a trend toward a stronger contribution of HLA-B-restricted CD8^+^ T cell responses to the total early HIV-1-specific CD8^+^ T cell response compared to HLA-A-restricted responses (HLA-B, 45% ± 39% versus HLA-A, 35% ± 36%; *p* = 0.08), in line with the above-described preferential restriction of frequently targeted epitopes (>50% of study participants) by HLA-B alleles (9/96 HLA-B epitopes tested versus 0/73 HLA-A epitopes; *p* < 0.01). HLA-C-restricted responses did not contribute significantly to the total detected HIV-1-specific CD8^+^ T cell responses, but the small number of described HIV-1-specific CD8^+^ T cell epitopes for these class C alleles [26] may have resulted in an underestimation of HLA-C-restricted responses, whereas a similar number of epitopes were tested for HLA-A and HLA-B (median of 7 epitopes per A allele, range 3–18; median of 8 epitopes per B allele, range 3–14; *p* = 0.6 [see Table 1]).

**Figure 3:**
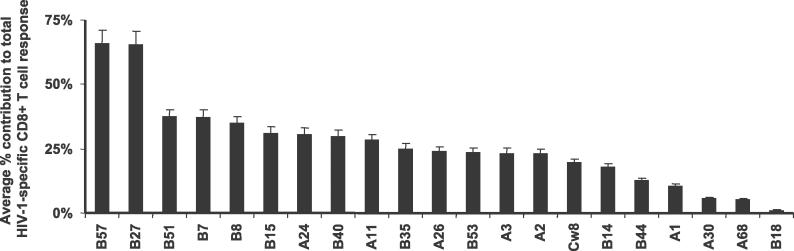
Different HLA Class I Alleles Differ in Their Contribution to the Total HIV-1-Specific CD8^+^ T Cell Response The percentage contribution of HIV-1-specific CD8^+^ T cell responses restricted by each individual HLA class I allele to the total HIV-1-specific CD8^+^ T cell response in individuals expressing the respective allele is shown. HLA class I alleles are listed according to their contribution from left to right. HLA-B57 and HLA-B27 contributed 66% and 65.4%, respectively, to the total HIV-1-specific CD8^+^ T cell response in individuals expressing these alleles.

In line with previous data [23], HIV-1-specific CD8^+^ T cell responses restricted by HLA-B57 contributed strongly to the total virus-specific immune response, accounting for an average of 66% of the total response against HIV-1 among individuals with this allele (Figure 3). HLA-B57 was followed by HLA-B27, another allele that has been associated with slower HIV-1 disease progression [27–29,36,37], in percent contribution to the total HIV-1-specific CD8^+^ T cell response (Figure 3). The robust contribution of these two alleles was not due to a higher number of epitopic peptides tested for these alleles (5 for HLA-B27 and 13 for HLA-B57 compared to a median of 6, range 3–18 for all the remaining alleles studied; *p* = 0.9), but was rather due to dominant responses to a single epitope in each case (TW10 for B57 and KK10 for B27, also see Figure 1, which shows a clear dominant response for each of these alleles).

**Figure 4:**
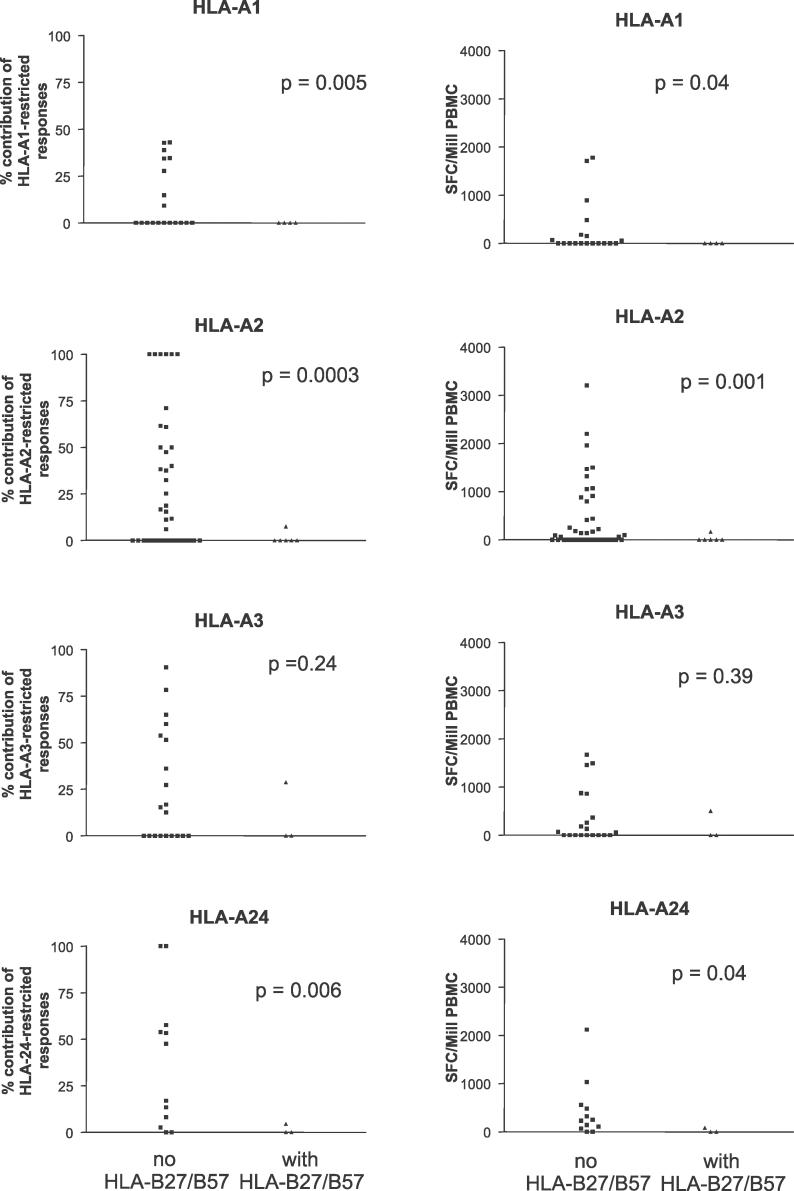
Immunodomination of HLA-B57- and HLA-B27-Restricted HIV-1-Specific CD8^+^ T Cell Responses The percent contribution (left graphs) and the absolute magnitude (right graphs, given as SFCs per million input PBMCs [SFC/Mill PBMC]) of HLA-A1-, -A2-, -A3-, and -A24-restricted HIV-1-specific CD8^+^ T cell responses in individuals expressing these HLA class I alleles alone, or in conjunction with HLA-B57 or HLA-B27, are shown. Each dot represents data for one individual. The contribution, as well as the absolute magnitude, of HIV-1-specific CD8^+^ T cell responses directed against HLA-A1-, -A2-, and -A24-restricted CD8^+^ T cell epitopes was significantly lower in participants that also coexpressed HLA-B57 or HLA-B27. The same trend was observed for HLA-A3, but did not reach statistical significance. HLA-A1-, -A2-, -A3-, and -A24-restricted HIV-1-specific CD8^+^ T cell response did not differ between individuals expressing other frequent HLA class B alleles, such as HLA-B7, -B8, -B35, or -B44 (unpublished data).

The dominant effect of HLA-B27 and -B57 in restricting HIV-1-specific CD8^+^ T cell responses during primary infection was further demonstrated by a significant difference in the contribution of other studied HLA alleles to the total response in the presence versus absence of HLA-B57 and -B27. The great diversity of HLA genotypes allowed this comparison for only a selected number of alleles that were sufficiently frequent in this study cohort. As exemplified in Figure 4 for the frequently expressed alleles HLA-A1, -A2, -A3, and -A24, the average contribution of these alleles to the total virus-specific CD8^+^ T cell response was 13% (HLA-A1), 27% (-A2), 25% (-A3), and 38% (-A24) in the absence of HLA-B57 and HLA-B27 expression. These contributions decreased dramatically in the presence of HLA-B57 or HLA-B27 (HLA-A1, 0%, *p* = 0.005; -A2, 1%, *p* < 0.001; -A3, 10%, *p* = 0.24; and -A24, 2%, *p* = 0.006; Figure 4), despite the fact that the same HLA-A1, -A2, -A3, or -A24 epitopes were tested under both conditions. This reduction in the relative contribution of HLA-A1-, -A2-, -A3-, or -A24-restricted HIV-1-specific CD8^+^ T cell responses was due to reduction in the absolute magnitude of virus-specific CD8^+^ T cell responses restricted by these HLA-A alleles in the presence of HLA-B57 and -B27 (Figure 4), and not to a relatively higher magnitude of responses restricted by the HLA-B alleles. These data suggest an active immunodomination of HLA-B57 and -B27-restricted CD8^+^ T cell responses during primary HIV-1 infection over other HLA allotypes. In contrast to these two HLA class I alleles, which are associated with slower disease progression, the presence of other HLA-B alleles frequently expressed in this study population, such as HLA-B44, -B35, -B8, and -B7, did not result in a detectable reduction of the relative or absolute HIV-1-specific CD8^+^ T cell response restricted by these HLA-A alleles (*p* > 0.1 for all comparisons, unpublished data).

### HLA Class I Allele-Specific Relative Hazard for Disease Progression Correlates Inversely with the Contribution to the HIV-1-Specific CD8^+^ T Cell Response

**Figure 5:**
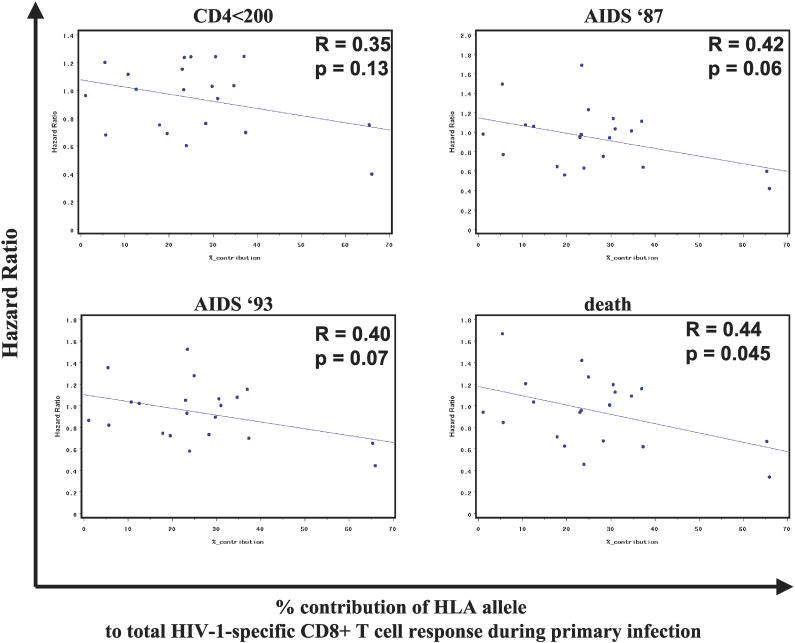
Correlation between the Contribution of Individual HLA Class I Alleles to the Total HIV-1-Specific CD8^+^ T Cell Response during Primary Infection and the HR for HIV-1 Infection Outcome The percent contribution of individual HLA class I alleles to the total HIV-1-specific CD8^+^ T cell response during primary infection was correlated to the HR for four different HIV-1 infection outcomes (time to CD4 <200, time to AIDS 1987, time to AIDS 1993, and time to death) for the respective HLA alleles.

The above data for HLA-B57 and HLA-B27 demonstrate that these two alleles, which have been strongly associated with slower HIV-1 disease progression, contribute substantially to the total HIV-1-specific CD8^+^ T cell response during primary HIV-1 infection. We next tested whether the contribution of an individual HLA class I allele to the total HIV-1-specific CD8^+^ T cell response in primary infection is associated with the HR for that allele in progression to four HIV-1 disease outcomes (CD4 <200, AIDS 1987, AIDS 1993, and death), using data from four HIV-1 cohorts (MACS, MHCS, SFCCC, and ALIVE) [27–30]. For all four outcomes, the contribution of an HLA allele to the HIV-1-specific CD8^+^ T cell response during primary infection showed a consistent inverse correlation with the respective HR (Figure 5), and this inverse correlation trended toward significance for the outcomes AIDS 1987 (*p* = 0.06), AIDS 1993 (*p* = 0.07), and reached significance for death (*p* = 0.045). While the correlation was strongly driven by HLA-B27 and HLA-B57, this analysis, which compared two discrete groups of patients, suggests that the ability of an HLA class I allele to dominate the HIV-1-specific T cell responses during primary infection might have an important impact on its protective role during the subsequent course of infection.

## Discussion

Several studies suggest that innate and adaptive immunological events occurring during primary HIV-1 infections play a crucial role in determining the level of viral replication during the subsequent disease process. The characterization of the individual epitopes frequently and consistently targeted by these early and potent virus-specific CD8^+^ T cells is therefore of major interest both for the identification of highly immunogenic targets for HIV-1 vaccines and for studies of the mechanisms underlying immunodominance in HIV-1 infection. Here we demonstrate in a cohort of 104 individuals with primary HIV-1 infection that only a subset of known CD8^+^ T cell epitopes is frequently and consistently targeted in the initial stages of HIV-1 infection, when viral load drops on average more than 1,000-fold. We found, furthermore, that the HLA class I genotype determines the level of contribution of CD8^+^ T cells responses restricted by individual alleles to the total HIV-1-specific T cell response. HLA alleles that contribute strongly to the initial virus-specific CD8^+^ T cell response represented those alleles associated with slower HIV-1 disease progression.

In the present study we used peptides corresponding to optimal HIV-1-specific CD8^+^ T cell epitopes to assess their frequency of recognition during primary HIV-1 infection. While the use of described optimal cytotoxic CD8^+^ T lymphocyte epitopes within HIV-1 may have resulted in an underestimation of total HIV-1-specific T cell responses, this approach allowed us to identify a subset of immunodominant epitopes that are very frequently targeted during this early phase of HIV-1 infection. In contrast, other well-characterized epitopes restricted by the same HLA class I alleles and located within the same HIV-1 proteins were only rarely recognized, demonstrating consistent immunodominance patterns of HIV-1-specific CD8^+^ T cell responses during primary HIV-1 infection. Furthermore, CD8^+^ T cell responses in primary infection were directed against a narrow repertoire of HIV-1 epitopes, suggesting that a limited number of CD8^+^ T cell responses are sufficient to control HIV-1 replication during the initial phase of infection. While these data do not allow for direct conclusions regarding the epitope-specific CD8^+^ T cell responses that need to be induced by vaccines in order to mediate protective immunity, they suggest that the presence of broadly directed and strong HIV-1 CD8^+^ T cell responses is not an absolute requirement for protective immunity.

Little is known about the mechanisms that determine immunodominant T cell responses in HIV-1 infection, but several factors—including the intracellular expression levels of the respective HIV-1 protein, the kinetics by which an epitope is processed, its binding affinity to the HLA class I molecule, and the binding affinity of the peptide–HLA class I complex to the CD8^+^ T cell receptor and its repertoire—have been implicated in the shaping of immunodominance patterns [38,39]. The current study did not allow us to address the individual contributions of these “intrinsic” factors to the observed hierarchies in epitope-specific CD8^+^ T cell responses. However, we demonstrate here that the sequence variability within HIV-1 is not a major factor in determining immunodominance pattern during primary HIV-1 infection, as frequently targeted epitopes did not significantly differ in their sequence variability within the HIV-1 population from rarely targeted epitopes restricted by the same HLA allele within this study cohort. Taken together, these studies of HIV-1-specific CD8^+^ T cell responses during primary HIV-1 infection on the single-epitope level resulted in the identification of several immunodominant epitopes within HIV-1 that are frequently targeted in infected individuals of Northern European descent.

HLA class I molecules are codominantly expressed on antigen-presenting cells, such that all six HLA class I allotypes (if heterozygous for HLA-A, -B, and -C) can present viral epitopes and theoretically prime virus-specific CD8^+^ T cell responses. However, epitopic peptides compete for presentation by HLA molecules, and different HLA class I molecules may differ in their ability to present the respective viral epitopes on the cell surface. Here we demonstrate that selected HLA class I molecules contribute significantly more than other HLA class I molecules to the total HIV-1-specific CD8^+^ T cell response detected during primary infection. Interestingly, the presence of HLA-B57 and HLA-B27, two alleles that contributed more than 65% to the total antiviral CD8^+^ T cell response among individuals carrying these alleles, reduced the relative contribution of HIV-1-specific CD8^+^ T cell responses restricted by other HLA molecules expressed in the same individual, as well as the absolute virus-specific CD8^+^ T cell response restricted by these other alleles. The large number of persons examined in this study allowed us to assess the impact of HLA-B27 and -B57 on the HIV-1-specific T cell responses restricted by other alleles, but the high HLA diversity limited this assay to the most frequent alleles, including HLA-A1, -A2, -A3, and -A24. For all four HLA-A alleles studied, the magnitude of HIV-1-specific CD8^+^ T cell responses restricted during primary infection was dramatically lower in the presence of HLA-B27 and -B57, but not in the presence of other HLA-B alleles. These data suggest a selective disadvantage of CD8^+^ T cell responses restricted by these alleles in the presence of HLA-B27 and -B57, and is consistent with the concept of immunodomination by HLA-B27- and HLA-B57-restricted HIV-1-specific CD8^+^ T cell responses [38].

Immunodomination in the evolution of dominant virus-specific CD8^+^ T cell responses, first studied in mice, refers to the ability of CD8^+^ T cells specific for immunodominant epitopes to suppress the CD8^+^ T cell response to subdominant epitopes. In these mouse studies, it was shown that the elimination of an immunodominant epitope resulted in the development of strong CD8^+^ T cell responses to otherwise subdominant epitopes [40–45]. Subsequent studies in humans demonstrated that immunodominant Epstein-Barr virus- and cytomegalovirus-specific CD8^+^ T cell responses restricted by HLA-A2 were reduced in those who coexpressed HLA-B7 [46,47]. Furthermore, simian immunodeficiency virus-infected rhesus macaques expressing both Mamu-A*01 and -A*02 were recently shown to exhibit a significant delay in the development of a CD8^+^ T cell response to a Mamu-A*02-restricted epitope that represented the immunodominant response in Mamu-A*02^+^ macaques in the absence of Mamu-A*01 expression [48]. Similar to HLA-B27 and -B57, two HLA alleles associated with slow disease progression in HIV-1-infected humans [27–29,36,37], Mamu-A*01 has been associated with slower disease progression in simian immunodeficiency virus-infected macaques [49–53], indicating that immunodomination by these major histocompatibility complex alleles in macaques and humans may contribute to their superior ability to control viral replication. Furthermore, these three alleles, which are associated with slower disease progression in HIV-1 infection, all restrict a single highly immunodominant epitope-specific CD8^+^ T cell response during primary infection (TW10 for B57 [23], KK10 for B27, and TL8 for Mamu-A*01 [8,51]), suggesting that the combination of these specific epitopes in the context of the restricting major histocompatibility complex class I allele may be crucial for the described strong antiviral activity. Further studies will be needed to elucidate the mechanisms underlying the immunodominance of virus-specific CD8^+^ T cell responses restricted by these alleles.

We tested directly for an association between the contribution of an individual HLA class I allele to the total virus-specific CD8^+^ T cell response in primary HIV-1 infection and its protective effect on disease progression. The average contribution of each studied HLA class I allele to the total HIV-1-specific T cell response detected using optimal CD8^+^ T cell epitopes was calculated in the 104 study participants, and correlated with the HR for the respective alleles for four different HIV-1 disease outcomes, as determined previously in the combined MHCS, ALIVE, MACS, and SFCCC cohort studies [27–30]. For each of the four outcomes considered (progression to CD4<200, AIDS 1987, AIDS 1992, and death), the antiviral contribution of an allele was negatively correlated with its HR, and this inverse correlation was borderline significant for the outcomes AIDS 1987 (*p* = 0.06), AIDS 1993 (*p* = 0.07), and death (*p* = 0.045). These data suggest that HLA class I alleles that contribute robustly to the initial CD8^+^ T cell response against HIV-1 delay progression to AIDS. It is notable that individuals in these different cohorts of HIV-1 infected participants were grouped according to low-resolution two-digit HLA class I typing, and this association was strongly driven by the associations for HLA-B57, HLA-B51, and HLA-B27. Future studies of larger cohorts that take high-resolution HLA class I subtypes into account will be needed to provide greater detail on the contribution of specific HLA class I subtypes to the total HIV-1-specific CD8^+^ T cell response and disease progression.

Taken together, these studies in a large cohort of individuals with primary HIV-1 infection demonstrate consistent immunodominance patterns of HIV-1-specific CD8^+^ T cell responses during primary infection and provide a mechanistic link for the observed protective effect of specific HLA class I alleles on HIV-1 disease progression. Understanding the precise factors that allow epitope-specific CD8^+^ T cell responses restricted by individual HLA class I alleles to dominate over other epitopes restricted by the same allele, as well as over epitope-specific T cell responses restricted by other alleles, will require additional studies and be an important step in efforts to modulate antiviral T cell responses generated by therapeutic immunizations or vaccinations.

## Abbreviations

ALIVE: AIDS Linked to Intravenous Experience
ELISPOT: enzyme-linked immunosorbent spot
HLA: human lymphocyte antigen
HR: hazard ratio
MACS: Multicenter AIDS Cohort Study
MHCS: Multicenter Hemophilia Cohort Study
PBMC: peripheral blood mononuclear cell
SFC: spot-forming cell
SFCCC: San Francisco City Clinic Cohort
